# Good outcomes of modified Grammont and Langenskiöld technique in children with habitual patellar dislocation

**DOI:** 10.1007/s00167-020-06284-y

**Published:** 2020-09-26

**Authors:** Bartosz Jan Musielak, Pirunthi Premakumaran, Piotr Janusz, Magda Dziurda, Aleksander Koch, Michał Walczak

**Affiliations:** 1 Department of Paediatric Orthopaedics and Traumatology, Poznan University of Medical Sciences, Poznan, Poland; 2 Poznan University of Medical Sciences, Poznan, Poland; 3 Department of Spine Disorders and Paediatric Orthopaedics, Poznan University of Medical Sciences, Poznan, Poland

## Abstract

**Purpose:**

In this study, the functional mid-term outcomes of the modified Grammont and Langenskiöld technique was assessed in skeletally immature patients with habitual patellar dislocation, with emphasis on knee function, pain, and other possible post-surgical complications. This is the first study concerning the application of the modified Grammont and Langenskiöld technique in habitual patellar dislocations.

**Methods:**

This retrospective cohort study considered 10 patients (15 knees), ranging from 7 to 11 years old, who underwent the modified Grammont and Langenskiold procedure between 2015 and 2018. History of dislocation, patellar stability and range of motion (ROM) were analysed. To assess functional improvement and knee pain, the Kujala Anterior Knee Pain Scale and KOOS-Child Knee Survey were used before and after surgical treatment.

**Results:**

No history of dislocation was noted after surgical treatment. All 15 knees showed full ROM. There were no signs of genu recurvatum and no length discrepancies were found. The subjective assessment revealed significant improvement in the scores of the KOOS-Child questionnaire in all five sections (*p* < 0.001), as well as in The Kujala Anterior Knee Pain Scale (*p* = 0.001).

**Conclusion:**

The modified Grammont and Langenskiöld technique yields remarkable results in terms of knee stability and knee function, while decreasing recurrence risk and intensity of pain in patients with challenging cases of patellofemoral joint dislocation. This surgical technique is most effective in cases where the patella remains dislocated continuously; however, it may also be used in immature patients with recurrent instability.

**Level of evidence:**

IV.

## Introduction

Habitual patellar dislocation in children is considered an orthopedic condition which is challenging to treat due to its complexity. There are several elements in this pathology which need to be addressed if one would like to achieve a positive outcome of treatment. These include patellar dislocation, trochlear groove dysplasia, tibial tuberosity lateralization, medial capsule elongation, and lateral capsule contracture [4, 7, 20, 27]. Conservative treatment is partially effective in patients with primary or recurrent patellar dislocation, however, this is inadequate in cases of habitual dislocation, whereby surgical procedures are more effective [1]. The current approach to treating patellar dislocations, including MPFL reconstruction, is deemed ineffective with habitual dislocations. To efficiently negate all the contributors simultaneously, these pathologies require a more complex approach [2, 9, 17]. Moreover, selected parameters [e.g. Tibial Tuberosity–Trochlear Groove distance (TT–TG)], measured meticulously to indicate particular surgical techniques to be implemented in patellar dislocations, are established based on the adult patients and, therefore, have no use in children and adolescents.

As a result, this has led many authors to search for an effective, yet simple method of realignment of the patellofemoral joint. When used separately, the proximal and distal realignment techniques have been ineffective in the management of habitual dislocation of the patella [8, 13]. Therefore, a combination of procedures involving proximal and distal reconstruction is recommended [2, 10, 15]. In their original versions, the Grammont and Langenskiöld procedures both require complete detachment of the distal insertion of the patellar ligament, and reattachment towards the medial position. Nevertheless, this may significantly disturb the growth of the tibial tuberosity and slow down the process of postoperative rehabilitation [10, 15]. Paley modified these surgical techniques to overcome the problem of congenital patellar instability in Congenital Femoral Deficiencies (CFD) [19]. Interestingly, this modified technique could successfully be used in cases of habitual patella dislocations, whereas other surgical techniques used to date have produced rather average outcomes [3, 12, 14, 16, 17, 25].

All the above has prompted us to report the modified version of the Grammont and Langenskiöld technique, which was previously described as part of the SUPERknee procedure [19]. The functional and radiographic outcomes of this technique in skeletally immature patients were also reported.

## Materials and methods

IRB Approval was provided by the Poznan University of Medical Sciences Bioethical committee.

A total of 15 knee joints were reviewed retrospectively in ten patients (9 females and 1 male) with habitual dislocation of the patella. These patients were surgically treated with the modified Grammont and Langenskiöld procedure between 2015 and 2018. All patients were skeletally immature (wide-open physes, including tibial tuberosity physis, recognised through knee radiographs; Tanner stage I and II) and did not have any patellofemoral joint correction before the procedure. All patients presented similar patterns of patellar dislocation (the patella dislocates whenever the knee is flexed and spontaneously relocates with extension of the knee), and dislocation was confirmed before the age of six in all cases [2]. In four out of the ten patients hyperlaxity was present (which was confirmed using the Beighton score). The patients’ average age was 9 years and 1 month (range 7–11 years) at the time of the final examination. Only the patients who were operated on at least 24 months prior to their final check-up (with an average of 25.83 months) were included in this study. Patients presenting with neurological impairment, syndromes (such as nail-patella or Down’s syndrome), as well as those who had previous knee procedures, were excluded to keep the study homogenous. During the follow-up period, we were unable to reach two patients (three knees) and, therefore, a final examination was not performed on them.

All patients were evaluated before the initial surgical treatment (as a routine preoperative evaluation; by the first author) and again at least 2 years following the first treatment by three examiners (the first author, a senior resident and an intern). We analysed patellar stability based on clinical evaluation, including J-sign and apprehension test. Anterior knee pain (patellar grind test) and ROM were subsequently evaluated. Leg length was assessed in a standing and supine position with the use of standardized tests. If any discrepancy was noted on a patient, the long-leg standing radiograph was performed. To assess functional limitations and subjective symptoms concerning the patellofemoral joint, we used the Kujala Anterior Knee Pain Scale [11]. Additionally, subjective assessment of the outcome was evaluated with the use of KOOS-Child Knee Survey before and after surgical treatment [18]. In terms of radiological evaluation, the antero-posterior and lateral radiographs of the knees, as well as the tangential radiogaphs of the patellofemoral joint were performed. These were used to determine the type of dislocation (full or partial dislocation), the specific location of the patella (alta, baja), and any other knee axial deformities.

### Surgical technique

**Fig. 1:**
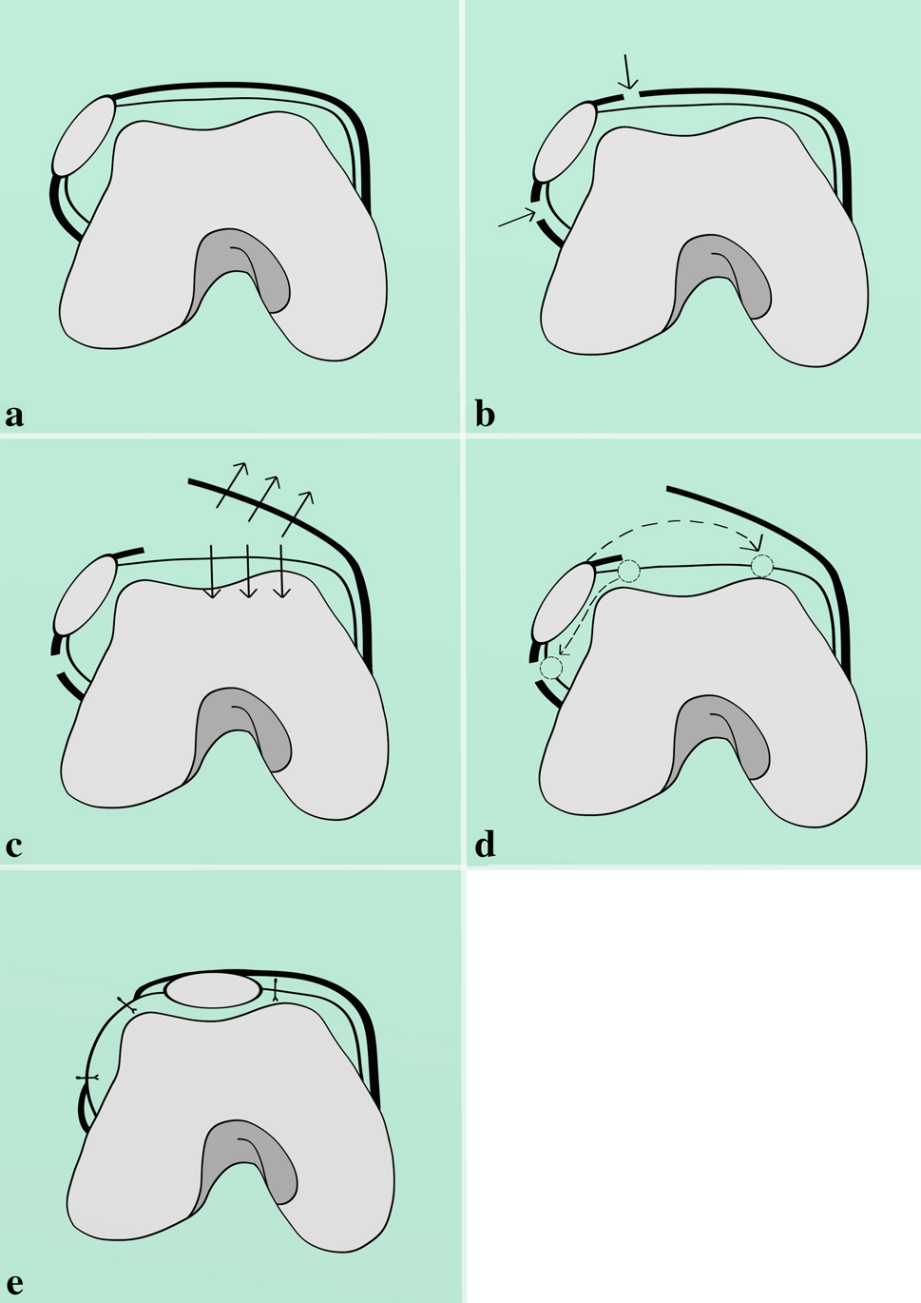
Modified Langenskiöld procedure—patella reduction: **a** axial view on the dislocated patellofemoral joint, thick line represents knee capsule, thin line—synovium; **b** knee capsule is detached from both sides of the patella; **c** knee capsule and the fascia are separated from the synovial membrane; **d** the patella is incised circumferentially out of synovium, new incision in the synovial membrane is performed above the sulcus, dashed arrow represents the direction of patella transposition; **e** the patella is attached to the new hole in the synovium, medial knee capsule is attached to the patella on its upper surface as laterally as possible, lateral capsule is left open

**Fig. 2:**
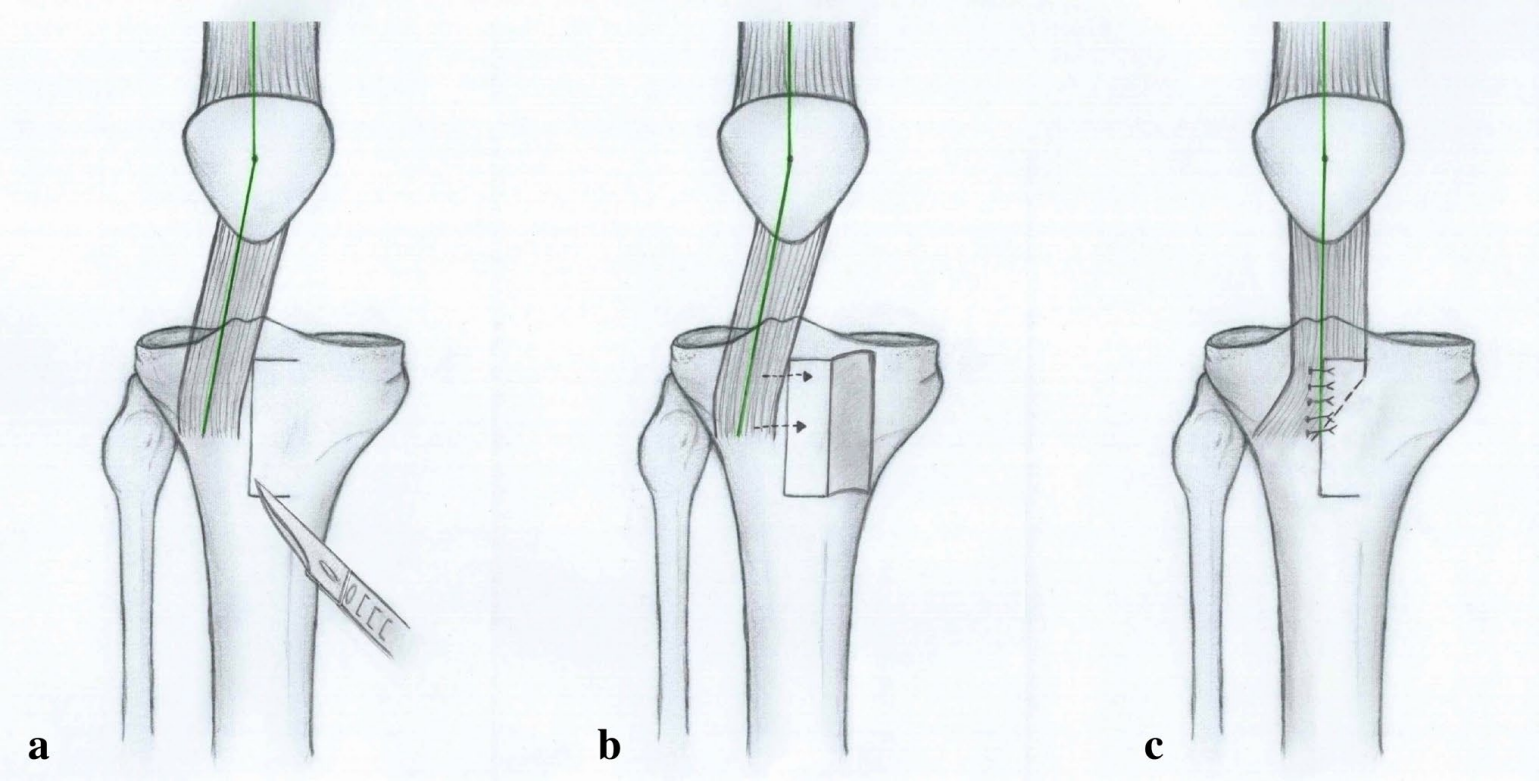
Modified Grammont procedure—medial transfer of the patellar ligament: **a** periosteal flap is created medially to the lateralised ligament; **b** ligament is transposed medially, without detachment of its insertion, under the elevated periosteal flap; **c** periosteal flap is sutured over the medialized ligament

The presented surgical technique consists of the Langenskiöld procedure, which is the reduction of the patella into the femoral groove by creating a new opening inside the synovium (Fig. 1), and the Grammont procedure, which is the medial transfer of the patellar ligament without detaching its distal insertion (Fig. 2) [19].

The capsule is incised and separated from the patella and synovium, medially and laterally (Fig. 1b). On the medial side, the two layers are separated all the way to the medial gutter (Fig. 1c). The knee capsule incision on the lateral side reaches the distal part of the patellar tendon, and up to the lower fibers of the vastus lateralis muscle, detaching them from the patella (Fig. 1b). The medial compartment of the capsule is cut close to the patellar rim, and extends down to the attachment of the patellar tendon to the tibial tuberosity (Fig. 1b).

In most of the cases, even though the capsule is separated from the patella at this stage of the surgery, the patella is not mobile enough due to the restraint coming from the contracted synovium. Thus, the synovium is then incised circumferentially around the patella (Fig. 1d), separating the patella from the synovium completely. The quadriceps tendon and patellar tendon remain attached to the patella proximally and distally, respectively.

The new incision in the synovium is performed above the femoral groove while ensuring its length correctly matches the size and level of the patella (Fig. 1d). The patella is now elevated from the synovium and placed in its new position over the femoral groove, and subsequently the synovium is sutured to the patella circumferentially (Fig. 1e). The empty hole in the synovium on the lateral side is sutured to restore its integrity and prevent a synovial fluid leakage (Fig. 1e).

Next, the maintenance of the patellar stability in knee motion and the Quadriceps angle (Q angle) are assessed. If the Q angle is greater than 20°, patellar ligament medialization would be performed (in this study—in all presented cases).

The patellar tendon is elevated from the tibial tuberosity and only the most distal attachment of the tendon remains intact (Fig. 2). The medial periosteum is usually elevated 1–1.5 cm medially (Fig. 2b).

The patellar tendon is then shifted medially and stabilized with non-absorbable sutures below the elevated periosteum, according to the actual position of the patella (Fig. 2b, c).

Finally, the medial capsule with the vastus medialis is advanced over the top of the patella and attached to its lateral border (Fig. 1e).

### Postoperative protocol

The limb is immobilized for 2 weeks in a cast, and is then replaced by a hinged knee brace. Passive motion is increased in 10° increments weekly, starting from 20°. Active knee extension and weight bearing of the operated limb are prohibited for 4 and 6 weeks, respectively.

### Statistical analysis

All the presented parameters were analyzed statistically with the use of non-parametric analysis (Wilcoxon signed-rank test). All statistical analysis were significant at *p* < 0.05 and were performed using GraphPad InStat software.

## Results

In all 15 knees the reduction of the patellofemoral joint (J-sign negative, lateralization of the patella absent throughout ROM) has been achieved, with no episodes of patellar dislocation during follow-up. In 1 knee, a patient maintained a positive apprehension test, while in the remaining 14 knees, the apprehension test was negative. One knee presented symptoms of anterior knee pain (patellar grind test positive). All 15 knees showed full ROM (with mean 0° of extension and 141.5° of flexion, which was symmetrical with the contralateral side) and no signs of genu recurvatum (knee hyperextension). Additionally, clinical and radiological evaluation of leg length did not reveal any discrepancy, or signs of patella alta (high-riding patella) and baja (low lying patella) between the sides in assessed patients.

**Fig. 3:**
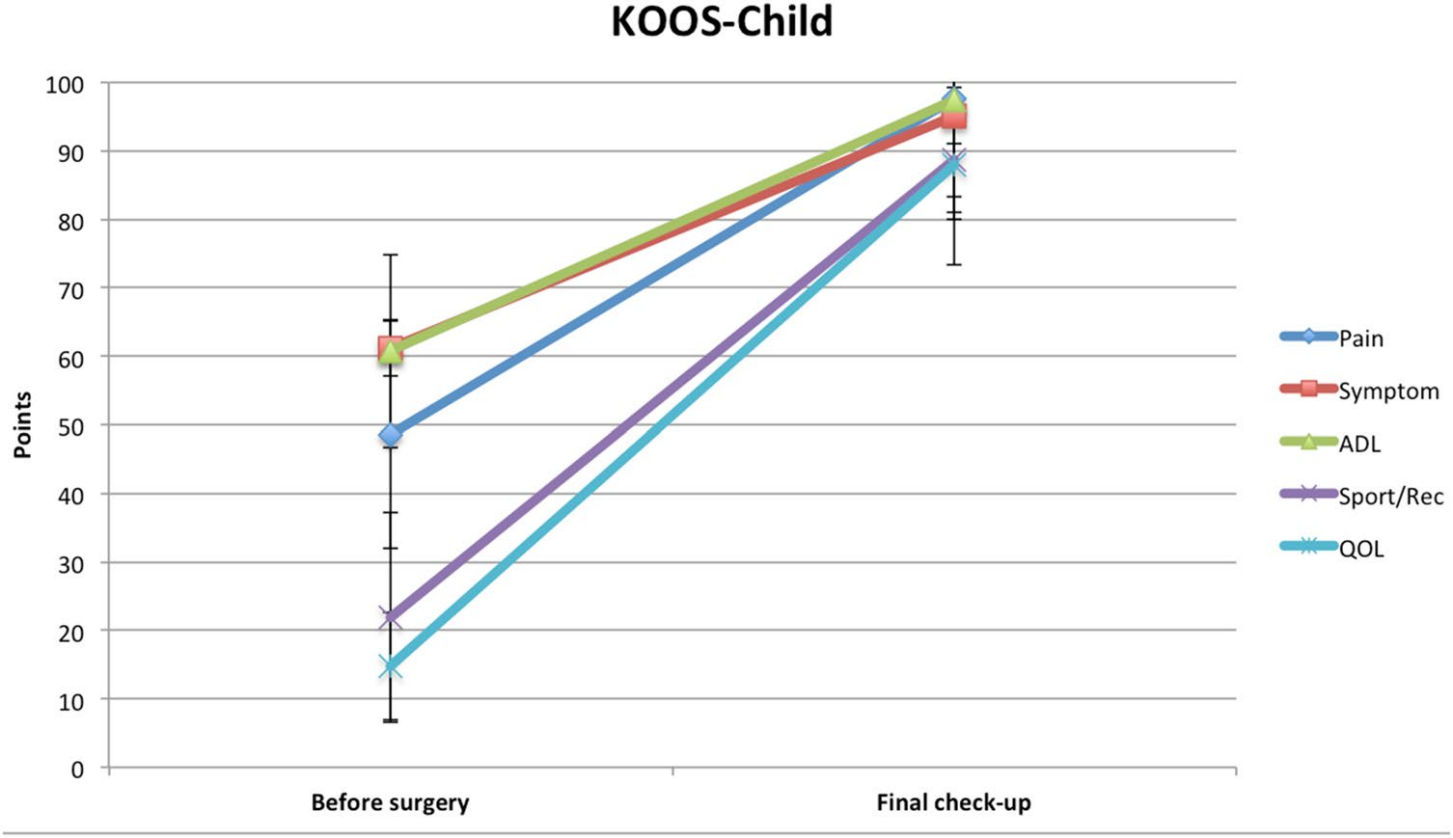
KOOS-Child Survey scores showing significant differences before surgical treatment and at the final check-up in each of the sections. *Symptom *other symptoms,* ADL *activities of daily living,* Sport/Rec *function in sport and recreation,* QOL knee-related Quality of life*

The subjective assessment of knee function revealed significant improvement in the scores of KOOS-Child questionnaire (Table 1) in all five sections (these being knee pain, other symptoms, the activities of daily living [ADL], physical activity, and quality of life [QOL]) (Fig. 3) [18]. Moreover, there was a significant improvement (*p* = 0.00096) in the Kujala Anterior Knee Pain Scale [11] in individuals operated on due to the habitual patellar dislocation (Table 2).

**Table 1:** Comparison of KOOS-Child Survey results in each section before surgical treatment and at the final check-up

	Mean	SD	*p* value
KOOS-Child pain			
Before surgery	48.6	18.6	0.0001
Final check-up	97.6	1.2	
KOOS-Child symptom			
Before surgery	61.2	5.2	0.0001
Final check-up	95.1	4.1	
KOOS-Child ADL			
Before surgery	60.8	14.1	0.0001
Final check-up	97.4	2.6	
KOOS-Child sport/rec			
Before surgery	21.9	16.3	0.0001
Final check-up	88.6	14.3	
KOOS-Child QOL			
Before surgery	14.8	8.8	0.0001
Final check-up	87.9	7.1

**Table 2:** Comparison of The Kujala Anterior Knee Pain Scale before surgical treatment and at the final check-up

		Before surgery	Final check-up
Kujala score	Mean	51.1	81.7
	SD	19.1	21.1
	*p* value	0.001	

## Discussion

The most important finding in this study was that in immature patients with habitual patellar dislocation, the modified Grammont and Langenskiöld technique provided permanent patella stability with proper tracking and no adverse effects such as decreased range of motion or growth impairment.

The treatment of habitual patellar dislocation in children is a complicated issue. The underlying anatomical pathology is complex and treatment should address patellar realignment, as well as extensors biomechanics [2, 9, 12, 21, 23]. In the pediatric population, this condition should be treated early, because it may progress to patellofemoral joint deformation, permanent pain, and osteoarthritis [23]. As conservative treatment of habitual patellar dislocation in young children is ineffective [21], the only possible treatment is surgical correction. However, the typical surgical methods used in adults are unfavorable in children, as they possess far more risks such as possible growth plate arrest and progressive genu recurvatum. Moreover, although the correction of one element brings very good results in recurrent patellar dislocation (e.g. MPFL reconstruction), it is usually insufficient in habitual patellar dislocation, as it requires more complex reconstruction [2, 9, 17]. Therefore, to obtain permanent reposition and good knee function in habitual patellar dislocation, a combination of proximal (patellar) and distal (tibial) reconstruction is recommended.

Based on obtained here results, the modified Grammont and Langenskiöld technique restores the correct anatomical conditions, provides adequate tracking of the patella, and minimizes the risk of growth plate arrest at the distal femur (which may be seen in other surgical techniques, e.g. when adversely locating the canal for MPFL graft) and the proximal tibia (e.g. when the patellar ligament’s distal insertion is completely detached and medialized) [14, 15]. Additionally, the creation of a new hole in the synovium for the patella is one of the parts of this technique that distinguishes it from the others. In the original technique described by Langenskiold, and later modified by Paley, the hole in the synovium was described as an essential part, however, the function of the synovium in the patellofemoral joint stability was not fully described [15, 19]. In our cohort, we observed that synovium is shortened on the lateral side. Although the synovium is not the main contributor to the dislocation, it seems to be contracted secondary to the dislocation. Therefore, without detaching it, we could not align the patella at the level of the groove. These observations, however, need to be studied further.

One of the most important issues concerning habitual dislocations is the recurrence risk. It varies in published data and is described to be between 0 and 22.9% depending on the study, procedure, and follow-up [3, 12, 14, 16, 17, 25]. According to Christensen et al. [5], the greatest contributing factor in recurrence is trochlear dysplasia, which causes an increase in sulcus angle and decrease in bony constraint of the patellofemoral joint. The patients in this study had a high risk of recurrent dislocation due to trochlear dysplasia (the sulcus was shallow or completely flat), soft tissue imbalance, and tuberosity lateralization. Despite this, within 2 years of follow-up, we have not noted a single recurrent dislocation in all observed patients (including one knee whereby the apprehension test was positive).

To perform the comprehensive evaluation of the knee treatment (apart from stability), subjective evaluation of the knee function and patients’ QOL is needed. To assess this, we used KOOS-Child Knee Survey and have noted an improvement with excellent results at 2 years follow-up in all five aspects of this survey (ADL, sports and recreation function, knee-related QOL, and knee-related pain and symptoms) [18, 22]. Moreover, the highest improvement was noted in QOL (from 14.8 to 87.9 points), and in sports and recreational function (from 21.9 to 88.6 points). On the other hand, the decrease of pain and knee-related symptoms, and improvement in daily living activities also bring the scores up to remarkable levels. When relating the results obtained here to what was published previously by Cootjans et al. [6], the modified Grammont and Langenskiöld technique gives the highest improvement in terms of QOL, sports activities, pain, and other knee-related symptoms compared to other surgical techniques. Meanwhile, other surgical techniques yield comparable results only in ADL [6, 17].

As for anterior knee pain and other knee-related symptoms, there was only one reported case (7.1%) with symptoms present (patellar grind test positive) during the clinical knee examination.

Furthermore, knee-related symptoms with Kujala Anterior Knee Pain Scale (a valid and reliable measure used for epidemiologic screening) were analyzed [11]. Two years after undergoing the modified Grammont and Langenskiöld procedure, Kujala score has increased notably by approximately 31 points (from 51.1 to 81.7). Comparison of these results to those obtained by other techniques previously published shows similar final outcome; however, most of the other studies do not present the scores before surgical correction, as presented in our research [12, 24, 26]. It must be emphasized that these comparisons are approximate, since the treated groups varied considerably in terms of age and severity of the instability.

Besides the retrospective design, the main limitation of this study is the limited cohort size due to the fact that this is a relatively rare condition. However, most of the published studies concerning this issue had similar or an even smaller number of patients, especially for habitual dislocations [3, 9, 12, 17]. Since our patients did not have an MRI done preoperatively, the indications for the patellar ligament realignment were arbitrary. However, this was not considered due to the following reasons: there is no existing norm of TT–TG distance for children, the patients showed no signs of intra-articular lesions, and MRI in children may require general anesthesia.

The other limitation is the follow-up period. However, 2 years is a sufficient time frame to reveal the most important complications related to growth, since all the patients were at their growing stage during the follow-up time. Finally, the control group was not presented as this was not the purpose of this study. Therefore, our results were compared to the results of other research concerning patella dislocations. Longer observation time and comparative study would be needed to draw a final conclusion.

## Conclusion

This is the first study concerning application of the modified Grammont and Langenskiöld technique in habitual patellar dislocations. This combined approach yields promising mid-term clinical results in terms of knee stability, recurrence risk, decrease in pain, and knee function in patients with challenging cases of patellofemoral joint dislocation. Moreover, this technique did not cause any changes to the limb axis, length, or the height of the patella within the observation period. Therefore, it may become an alternative for the most popular realigning techniques in immature patients such as the MPFL reconstruction with distal realignment.

## References

[1] Arendt EA, Fithian DC, Cohen E (2002). Current concepts of lateral patella dislocation. Clin Sports Med.

[2] Batra S, Arora S (2014). Habitual dislocation of patella: a review. J Clin Orthop Trauma.

[3] Benoit B, Laflamme GY, Laflamme GH (2007). Long-term outcome of surgically-treated habitual patellar dislocation in children with coexistent patella alta. J Bone Joint Surg Br.

[4] Biyani R (2014). Anatomical factors influencing patellar tracking in the unstable patellofemoral joint. Knee Surg Sports Traumatol Arthrosc.

[5] Christensen TC, Sanders TL, Pareek A (2017). Risk factors and time to recurrent ipsilateral and contralateral patellar dislocations. AMJ Sports Med.

[6] Cootjans K, Dujardin J, Vandenneucker H (2019). A surgical algorithm for the treatment of recurrent patellar dislocation. Results at 5 year follow-up. Acta Orthop Belg.

[7] Dejour H, Walch G, Nove-Josserand L (1994). Factors of patellar instability: an anatomic radiographic study. Knee Surg Sports Traumatol Arthrosc.

[8] Fondren FB, Goldner JL, Bassett FH (1985). Recurrent dislocation of the patella treated by the modified Roux-Goldthwait procedure. A prospective study of forty-seven knees. J Bone Joint Surg Am.

[9] Gao GX, Lee EH, Bose K (1990). Surgical management of congenital and habitual dislocation of the patella. J Pediatr Orthop.

[10] Grammont PM, Latune D, Lammaire IP (1985). Treatment of subluxation and dislocation of the patella in the child. Elmslie technique with movable soft tissue pedicle (8 year review). Orthopade.

[11] Ittenbach RF, Huang G, Barber Foss KD (2016). Reliability and validity of the anterior knee pain scale: applications for use as an epidemiologic screener. PLoS ONE.

[12] Joo SY, Park KB, Kim BR (2007). The ‘four-in-one’ procedure for habitual dislocation of the patella in children. J Bone Joint Surg Br.

[13] Kocon H, Kabacyj M, Zgoda M (2012). The results of the operative treatment of patellar instability in children with Down’s syndrome. J Pediatr Orthop B.

[14] Kraus T, Lidder S, Švehlík M (2012). Patella re-alignment in children with a modified Grammont technique. Acta Orthop.

[15] Langenskiöld A, Ritsilä V (1992). Congenital dislocation of the patella and its operative treatment. J Pediatr Orthop.

[16] Letts RM, Davidson D, Beaule P (1999). Semitendinosus tenodesis for repair of recurrent dislocation of the patella in children. J Pediatr Orthop.

[17] Mittal R, Balawat AS, Manhas V (2017). Habitual patellar dislocation in children: results of surgical treatment by modified four in one technique. J Clin Orthop Trauma.

[18] Örtqvist M, Roos EM, Broström EW (2012). Development of the knee injury and osteoarthritis outcome score for children (KOOS-Child). Acta Orthop.

[19] Paley D, Standard S, Flynn JM, Wiesel SW (2012). Treatment of congenital femoral deficiency. Operative techniques in orthopaedic surgery.

[20] Panni AS (2011). Patellar shape can be a predisposing factor in patellar instability. Knee Surg Sports Traumatol Arthrosc.

[21] Paton RW, Bonshahi AY, Kim WY (2004). Congenital and irreducible non-traumatic dislocation of the patella—a modified soft tissue procedure. Knee.

[22] Roos EM, Roos HP, Lohmander LS (1998). Knee injury and osteoarthritis outcome score (KOOS)—development of a self-administered outcome measure. J Orthop Sports Phys Ther.

[23] Sanders TL, Pareek A, Johnson NR (2017). Patellofemoral arthritis after lateral patellar dislocation: a matched population-based analysis. Am J Sports Med..

[24] Sillanpää P, Mattila VM, Visuri T (2008). Ligament reconstruction versus distal realignment for patellar dislocation. Clin Orthop Relat Res.

[25] Vähäsarja V, Kinnunen P, Lanning P (1995). Operative realignment of patellar malalignment in children. J Pediatr Orthop.

[26] Wegmann H, Würnschimmel C, Kraus T (2017). Medial patellofemoral ligament (MPFL) reconstruction in combination with a modified grammont technique leads to favorable mid-term results in adolescents with recurrent patellofemoral dislocations. Knee Surg Sports Traumatol Arthrosc.

[27] Zimmermann F, Liebensteiner MC, Balcarek P (2019). The reversed dynamic patellar apprehension test mimics anatomical complexity in lateral patellar instability. Knee Surg Sports Traumatol Arthrosc.

